# The mediating effects of self-efficacy and study engagement on the relationship between specialty identity and career maturity of Chinese nursing students: a cross-sectional study

**DOI:** 10.1186/s12912-024-02002-y

**Published:** 2024-05-21

**Authors:** Yanjia Liu, Mei Chan Chong, Yanhong Han, Hui Wang, Lijuan Xiong

**Affiliations:** 1 Department of Nursing, Wuhan Union Hospital, Tongji Medical College, Huazhong University of Science and Technology, Wuhan, China; 2 Department of Nursing Science, Faculty of Medicine, Universiti Malaya, Kuala Lumpur, Malaysia; 3 Department of Medicine, Jingchu University of Technology, Jingmen, Hubei China

**Keywords:** Nursing students, Self-efficacy, Study engagement, Specialty identity, Career maturity, Mediating effect

## Abstract

**Background:**

Career maturity is a crucial indicator of career preparedness and unpreparedness can cause the turnover of new nurses. Considerable empirical work demonstrates the potential associations between specialty identity, self-efficacy, study engagement, and career maturity. This study aimed to explore the mediation role of self-efficacy and study engagement on the relationships between specialty identity and career maturity among Chinese nursing students.

**Methods:**

Four hundred twenty-six Chinese nursing students were recruited between September 11 and October 30, 2022. The online survey was conducted following the CHERRIES checklist. Electronic questionnaires assessed their perceived specialty identity, self-efficacy, study engagement, and career maturity. The descriptive analysis, Harman single-factor analysis, Pearson correlation tests, structural equation modeling, and the bootstrap method were employed in data analysis.

**Results:**

Bivariate correlation analysis identified a positive correlation between specialty identity, self-efficacy, study engagement, and career maturity (*r* = 0.276–0.440, *P* < 0.001). Self-efficacy and study engagement partially mediated the relationship between specialty identity and career maturity. Self-efficacy and study engagement played a chain mediating role between specialty identity and career maturity.

**Conclusions:**

The underlying mechanism can explain the relationships between specialty identity and career maturity: a direct predictor and an indirect effect through self-efficacy and study engagement. Policymakers and educators should emphasize the importance of specialty identity and provide tailored strategies for improving care maturity depending on nursing students’ specialty identity, self-efficacy, study engagement in the early stages of career development.

**Supplementary Information:**

The online version contains supplementary material available at 10.1186/s12912-024-02002-y.

## Introduction

The media visibility obtained by nursing during the COVID-19 pandemic has made the public aware of nurses’ role in promoting and maintaining health [1]. As the social environment becomes more conducive to nursing career development, adequate awareness and preparedness for nursing careers are driving nursing students to adapt to and be satisfied with their careers [2, 3].

Career maturity is a crucial indicator of career preparedness [4], which is defined as the readiness to make age-appropriate career decisions with adequate information and accomplish career development-related tasks [5]. Unpreparedness and difficulties in taking on the nurse’s role were the main reasons newly graduated nursing students left nursing in their first years [6]. The turnover rate for new nurses in their first year of employment can reach as high as 69%, with a range of 12.10–69% [6–9]. In addition, new nurses who experienced higher levels of career maturity were also less likely to leave the profession [10]. Therefore, more research focusing on career maturity should re-engage nursing educators and managers and support the development of customized programs in the early stage of career development.

## Background

### Specialty identity as a predictor to career maturity

Super’s theory emphasizes that career development is a lifelong activity closely related to individual maturity and experiences [11]. It encompasses the development of behaviors and professional identity [12]. Work values, including professional identity, are crucial for career development and can influence career maturity [13]. professional identity significantly correlates with high school students’ career maturity [14]. Additionally, specialty identity appears as a part of professional identity in studies worldwide [15]. To clarify this concept in student groups, specialty identity is defined as the emotional acceptance and recognition of learners based on their understanding of the specialty being studied, accompanied by positive external behaviors and an inner sense of satisfaction [15]. Therefore, this study proposes Hypothesis 1: specialty identity significantly predicts career maturity among Chinese nursing students.

### The mediating effect of self-efficacy between specialty identity and career maturity

Self-efficacy and career maturity are positively related [16, 17]. According to social cognitive theory, self-efficacy is a belief in a person’s ability to achieve their goal [18]. Regarding career maturity, self-efficacy could be an internal driver for students to dedicate themselves to the fields they have chosen [16]. Students with high self-efficacy can improve their professionalism and self-confidence, thereby achieving high degrees of career maturity [16]. Further, professional identity is found to be significantly correlated with self-efficacy [19, 20]. Yao et al. [21] found that self-efficacy mediated between professional identity and self-reported competence among nursing students. Thus, this study poses Hypothesis 2: Self-efficacy is the mediating variable affecting specialty identity and career maturity among Chinese nursing students.

### The mediating effect of study engagement between specialty identity and career maturity

Study engagement is a vital variable related to academic performance, achievement, persistence, and retention, which refers to a positive psychological process including attention, energy and effort in learning [22, 23]. Astin’s theory of student involvement emphasizes that the significant environmental factors that can influence their engagement entail students’ backgrounds, such as residence, experiences, and academic involvement [24]. A significant correlation exists between study engagement and career maturity [25]. Moreover, Liu et al. [26] report that professional identity is positively correlated with study engagement, and the mediating role of study engagement in professional identity and career adaptability is significant. Based on the above evidence, this study posits Hypothesis 3: study engagement is the mediating variable affecting specialty identity and career maturity among Chinese nursing students.

### The chain mediating effect of self-efficacy and study engagement between specialty identity and career maturity

Based on the aforementioned information, self-efficacy and study engagement may play a single mediating role between specialty identity and career maturity. However, the relationship between self-efficacy and study engagement remains to be clarified. In addition, whether these variables play a chain mediating effect between specialty identity and career maturity must be explored. Previous research has shown that self-efficacy positively correlates with study engagement [27, 28]. The relationship between specialty identity and career maturity may be influenced by self-efficacy in the first place and by study engagement in the second. Therefore, this study proposes Hypothesis 4: Chain mediation describes the relationship among the four variables.

Overall, this study explores the relationship between specialty identity and career maturity. It also examines the potential mediation model of specialty identity, self-efficacy, and career maturity, the potential mediation model of specialty identity, study engagement, and career maturity, and the potential chain mediation of the four variables using mediation analysis.

## Methods

### Design

This cross-sectional online survey was conducted among nursing students between September 11 and October 30, 2022. This online survey was designed, disseminated and conducted following the Checklist for Reporting Results of Internet E-Surveys (CHERRIES) [29] (see in the Supplement File 1). The online questionnaire entailed demographic sheet and four instruments with different question styles (single choice and Likert scales).

### Variables and data collection instruments

#### Sociodemographic variables

Sex (female, male), Higher education institution type (university, college), and Degree (diploma, bachelor’s).

#### Specialty identity

The College Student Specialty Identity Scale (CSSIS) developed by Qin [15], was used to measure medical students’ specialty identity [30]. It is a 23-item scale with four subscales (cognitive, emotional, behavioral, and appropriateness). Students scored each item on a five-point Likert scale (1–5: strongly disagree to strongly agree). In Qin’s study, it had good reliability (α = 0.955) [15]. In the present study, Cronbach’s α was 0.949.

### Study engagement

Study engagement was assessed using the Utrecht Work Engagement Scale-Student (UWES-S) [31]. Schaufeli et al. [32] developed the UWES and revised its items to measure students’ study engagement. Li & Huang [31] introduced UWES-S, translated it into Chinese, and validated it among undergraduate students. The UWES-S comprises 17 items grouped into three subscales (vigor, dedication, and absorption) and uses a 7-point scale (0 = never, to 7 = always). The cumulative scores range from 0 to 102, with higher scores indicating greater study engagement. The internal consistency using Cronbach’s alpha was 0.919 [31]. In this study, Cronbach’s α = 0.956.

### Self-efficacy

This study used the Chinese version of the General Self-Efficacy Scale to assess self-efficacy [33]. Schwarzer et al. [34] developed the original version. It was adapted to the context of China and validated by Wang et al. [33]. It is a 10-item scale with a 4-point Likert scale (from completely incorrect to completely correct). Cronbach’s alpha was 0.871 in Wang et al.’s study [33]. In this study, Cronbach’s α = 0.899.

### Career maturity

Career maturity was measured using the validated Chinese version of the career maturity scale [35]. The original version developed by Lee [36] was translated into Chinese by Zhang et al. [35]. The instrument entails 34 items broken down into six subscales: career decisiveness (CD), career confidence (CC), career independence (CI), career value (CV), relational dependence (RD), and career reference (CR). A 5-point Likert scale, ranging from strongly disagree to strongly agree, was adopted. The range of total scores was 34–170, with higher values indicating higher levels of career maturity. Its reliability coefficient was 0.86, as measured using Cronbach’s alpha [35]. In this sample, Cronbach’s α = 0.900.

### Participants and data collection procedure

This study was conducted at five higher education institutions in Hubei province, China. The target population, full-time nursing students, were surveyed using convenience sampling. The suggested minimum sample size based on Monte Carlo simulations studies was adopted [37], and the minimum and maximum sample sizes for structural equation models were 200 and 460 respectively [38]. The final sample size in the design stage was 250–575, accommodating a possible dropout rate of 20%.

For data collection, we uploaded the integrated questionnaires on Wenjuanxing (https://www.wjx.cn/, Acquired NO.168,902,709). This website offers the most popular and convenient tool for anonymous data collection and collecting data anonymously extraction. One investigator from each university or college was invited to collect the data. All investigators held master’s degrees and understood the critical points for questionnaire collection well. Simple training was conducted before questionnaire distribution. Each nursing student can review and change their answers if necessary, but they were provided only one chance to submit the online questionnaires. The individual IP address can be yielded after submissions and provided for verification. Finally, 560 questionnaires were administered. After double-checking and eliminating invalid questionnaires, 426 valid questionnaires were extracted, yielding an effective response rate of 76.07%.

### Data analysis

After completing the descriptive analysis, Harman single-factor analysis was performed to assess the common method bias, and Pearson correlations were calculated in SPSS Version 26.0. Subsequently, structural equation modeling was validated, and the chain-mediation effect was examined using the bootstrap method in AMOS Version 23.0 with 5000 samples. The significance level was set at 0.05.

### Ethical considerations

This study was approved by the Ethics Committee of Jingmen No. 2 People’s Hospital, affiliated to Jingchu University of Technology (Approval No.2020002-1). All students provided verbal consent to participate in the study and voluntarily completed and submitted the questionnaire.

## Results

### Harman single-factor analysis

The self-reported nature of the data meant the possibility of common method bias [39]. The Harman single-factor analysis showed that the eigenvalues of the five common factors were greater than 1. The first common factor explained 35.50% of the variance, which is lower than the recommended threshold of 50% [40]. Therefore, no common method bias was detected.

### Descriptive statistics and correlation analysis

The sociodemographic variables were as follows: female (*n* = 370, 86.85%), male (*n* = 56, 13.15%); university (*n* = 322, 75.59%), college (*n* = 104, 24.41%); freshmen (*n* = 101, 23.71%), sophomores (*n* = 166, 38.97%), juniors (*n* = 127, 29.81%), seniors (*n* = 32, 7.51%); Urban areas (*n* = 151, 35.45%), Rural areas (*n* = 275, 64.55%). The age of nursing students range from 18 to 25 (mean = 19.89, SD=1.27 ) (see Table 1).

**Table 1:** Nursing students’ characteristics (*n* = 426)

Variables	*n*(%)/mean ± SD
Age	19.89 ± 1.27
Gender	
Male	56 (13.15)
Female	370 (86.85)
School	
University	322 (75.59)
College	104 (24.41)
Grade	
Grade 1 (freshmen)	101 (23.71)
Grade 2 (sophomores)	166 (38.97)
Grade 3 (juniors)	127 (29.81)
Grade 4 (seniors)	32 (7.51)
Home location	
Urban areas	151 (35.45)
Rural areas	275 (64.55)

Table 2 shows the mean scores of the four key variables were 80.40 ± 21.66, 57.56 ± 16.04, 26.23 ± 9.37, and 113.42 ± 31.61. Positive correlations were found between the key variables: specialty identity and study engagement (*r* = 0.276), specialty identity and self-efficacy (*r* = 0.319), specialty identity and career maturity (*r* = 0.300), study engagement and self-efficacy (*r* = 0.420), study engagement and career maturity (*r* = 0.319), and self-efficacy and career maturity (*r* = 0.440) (each *p* < 0.001).

**Table 2:** Descriptive statistics and correlation analysis between key variables (*n* = 426)

				Correlations		
Variables	M	D	Specialty identity	Study engagement	Self-efficacy	Career maturity
Specialty identity	80.40	21.66	1			
Study engagement	57.56	16.04	0.276**	1		
Self-efficacy	26.23	9.37	0.319**	0.420**	1	
Career maturity	113.42	31.61	0.300**	0.319**	0.440 **	1

Note. ** *p* < 0.001

### The chain-mediation effect analysis

**Fig. 1:**
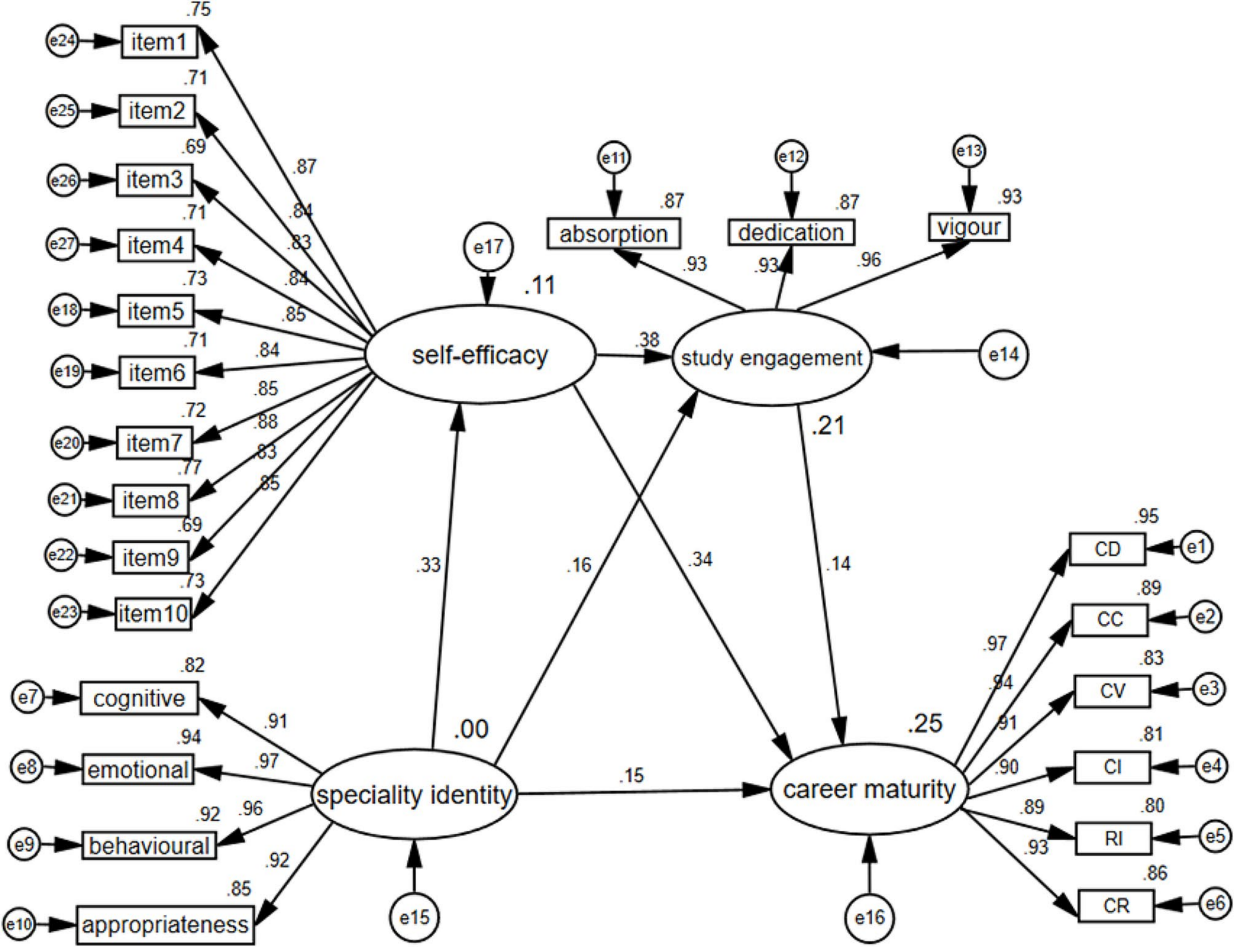
The chain mediation effect model diagram of specialty identity, self-efficacy, study engagement, and career maturity of nursing students

A chain-mediation structural model was constructed with specialty identity as the independent variable, career maturity as the dependent variable, and self-efficacy and study engagement as the mediating variables. The model fitting results showed that χ^2^/df = 2.965, the comparative fit index = 0.963, the Tucker-Lewis index = 0.958, and the root mean square error of approximation = 0.068, indicating good model fit. The chain-mediation effect model diagram of specialty identity, self-efficacy, study engagement, and career maturity of nursing students is shown in Fig. 1.

The bootstrapping method found that the 95% confidence interval (95% CI) of the chain-mediation path from specialty identity to career maturity was [0.002, 0.054], which did not include 0, indicating significance. Thus, two possible mediation effects were detected: the mediating roles of self-efficacy in the relationship between specialty identity and career maturity and of study engagement in the relationship between specialty identity and career maturity, both of which were significant with a 95% CI (see Table 3).

**Table 3:** Bootstrap analysis of the significance test of mediation effects

Effect	Pathway	Effect value	Effect Ratio(%)	95% CI	
				Low	High
Indirect effect	SI → self-efficacy → CM	0.155	35.38	0.101	0.233
	SI → SE → CM	0.031	7.08	0.002	0.085
	SI → self-efficacy → SE → CM	0.023	5.25	0.002	0.054
Direct effect		0.209	47.71	-	-
Total effect		0.438	100	0.264	0.618

Note. CI = confidence interval, SI = specialty identity, SE = study engagement, CM = career maturity

## Discussion

This study explored the relationships between specialty identity, self-efficacy, study engagement, and career maturity and demonstrated the mediation models in Chinese nursing students. The finding identified a positive correlation between specialty identity and career maturity, and specialty identity can influence career maturity in three ways: self-efficacy, study engagement, and self-efficacy → study engagement, supporting the four Hypotheses. Despite this study did not validate the potential confounders such as career resilience [20], career adaptability [26], and resource management [29], the findings may improve our understanding of the underlying mechanism of these four variables and provide meaningful ideas for taking measures to improve nursing students’ career maturity.

In this study, the findings revealed a positive correlation between specialty identity and career maturity, indicating that specialty identity could significantly predict career maturity in nursing students, which is consistent with previous studies [14, 41]. However, most nursing students enrolled in nursing school with insufficient specialty identity owing to poor nursing image and a lack of acknowledgment of career growth [42], meaning their unpreparedness for learning nursing and career development. Specialty identity is an emotional foundation of career maturity, and it can serve as a powerful psychological adjustment when it comes to nursing students’ specialty or job selection. Therefore, the importance of specialty identity on career maturity should be valued by nurse educators and clinical mentors, and further studies should specifically develop and conduct the education program to verify the roles of specialty identity in the early stage of career development. For example, an innovative course about the power of nursing including embracing the healer’s art course, seed talk and reflection exercises was found to connect the nursing students’ values to their specialty identity, and facilitate their professional formation and the development of nursing practice [43].

The first pathway confirmed was the mediating role of self-efficacy in the relationship between specialty identity and career maturity, aligning with its mediating effect on professional identity and career maturity in a previous study [41]. When nursing students perceive higher levels of specialty identity, they may have a stronger sense of self-efficacy and achieve greater career maturity. This finding is consistent with the Knowledge–Attitude–Belief–Practice model [44]. For nursing students, specialty identity and self-efficacy can support attitudes and beliefs about learning nursing specialties [21] and play the role of internal driving strength in the chase for a feasible professional study plan and career plan. As a result, career maturity could be a feedback indicator for learning behaviors and career preparedness.

This study also verified the mediating effect of study engagement on specialty identity and career maturity. This finding is consistent with a mediation analysis confirming the mediating role of study engagement between professional identity and career maturity among pre-service kindergarten teachers [25]. This result also supports the predictive impact of study engagement on the beneficial development of careers [26, 45]. The mediating effect of study engagement revealed that if nursing students perceive high levels of specialty identity, they might have greater study engagement, achieve more knowledge and skills related to the nursing specialty, and possess high degrees of career maturity to adapt to the nursing profession. However, this study identified the study engagement had a limited mediating effect with a low effect size. The possible reason is that nursing is a specialized and complex discipline, which requires lifelong learning as health needs change and medical technology advances. In a short period, study engagement can improve nursing knowledge and skills, which is conducive to career preparedness, but high levels of career maturity are the result of long-term study engagement especially since this career needs continued education or continued career development [46].

Additionally, these findings supported the assertion that the chain relationship between self-efficacy and study engagement mediates the relationship between specialty identity and career maturity. The indirect effect of the pathway, including self-efficacy, was greater than that of the chain pathway and the pathway, including a single study engagement. Higher specialty identity could yield higher self-efficacy [19, 20], and higher self-efficacy is related to greater study engagement [27, 28]. Thus, nursing students with higher specialty identity might have higher self-efficacy and greater study engagement, which leads to higher career maturity. This model also revealed that increased self-efficacy might contribute to nursing students’ high study engagement levels. When nursing students have a sense of high self-efficacy, their learning behaviors become more effective. They are more willing to devote themselves to learning, thus producing higher study engagement. Despite some studies have demonstrated the effect of interventions such as career planning group counseling [47] and self-reflection-focused career course [48] on nursing students’ career maturity, what we found in this study provide theoretical foundation for the development and implementation of multifaceted interventions to improve nursing students career maturity and career development. Furthermore, further attention should be given to the interdisciplinary collaborations, such as positive psychology and nursing education, that can be contribute to explore novel perspectives and approaches to studying career maturity.

This study had some limitations. First, the cross-sectional design without a longitudinal method fails to explore the changes in psychological variables over time, which might restrict the temporal and causal inference. Therefore, scholars should focus on exploring the trajectory changes of these variables, notably the mutability of these psychological features, in the further studies, and the longitudinal and sustained interventions like tutor systems and peer learning should be strongly encouraged. Second, the nursing students were selected from five schools in Hubei province, China, which might limit the generalizability to all Chinese nursing students. As there are disparities in the curriculum systems of different schools, the results could be impacted by cognitive errors caused by teaching philosophy and training purposes. Therefore, the potential influencing factors should be considered and other mediators excluding self-efficacy and study engagement also should be explored in further studies. Third, selection bias may arise from the application of convenience sampling. Therefore, scholars could employ probability sampling methods like random stratified sampling to recruit nursing students. Finally, since all instruments were self-reported, the true feelings of these nursing students were not captured or tracked. From this, the research designs to deepen the understanding of the mechanisms underlying nursing students’ career development, such as mixed-method study and qualitative study, should be considered.

## Conclusion

A correlational and mediation analysis was used to examine the relationships between four variables. Specialty identity could be a predictive factor for nursing students’ career maturity. Most importantly, specialty identity can indirectly influence career maturity among nursing students through the mediating effect of self-efficacy, study engagement, and the chain mediating effect of self-efficacy and study engagement, supporting career-related theories. Policymakers and educators should focus on the value of specialty identity to promote nursing students’ career development. Specialty identity may be conducive to stimulating students with a strong sense of self-efficacy and robust study engagement. Nursing students with high self-efficacy and study engagement may perceive greater career maturity. Thus, scholars and educators should be encouraged to provide tailored career guidance programs and practical interventions to enhance nursing students’ career maturity in the early stage of career development.

### Electronic supplementary material

Below is the link to the electronic supplementary material.

Supplementary Material 1

## Data Availability

The datasets used and/or analyzed during the current study are available from the corresponding author on reasonable request.
